# Efficacy and safety of *Cyperus rotundus* extract on weight management in obese individuals: A randomized, double-blind, placebo-controlled study

**DOI:** 10.1097/MD.0000000000045666

**Published:** 2025-11-21

**Authors:** Anju Majeed, Shaheen Majeed, T. V. Devarajan, S. S. V. V. Narasinga Rao, Satish Gudimallam, Manjunatha Ramanujappa, Smitha Thazhathidath, Lakshmi Mundkur

**Affiliations:** a Sami-Sabinsa Group Limited, Bengaluru, Karnataka, India; b Sabinsa Corporation, East Windsor, NJ; c Apollo First Med Hospitals, Chennai, India; d Govt. Medical College and Govt. General Hospital (Old RIMSGGH), Srikakulam, India.

**Keywords:** Apolipoprotein B, *Cyperus rotundus*, lipid levels, obesity, stilbenes

## Abstract

**Background:**

Obesity, a critical public health issue, is linked to numerous metabolic and cardiovascular disorders. Natural products may present a safer and more holistic approach to weight management. A standardized extract of *Cyperus rotundus* (CRE), containing 6% to 8% stilbenes (scirpusin A, scirpusin B, and piceatannol) was effective in reducing adipogenesis and weight gain in preclinical studies. This study aimed to assess the efficacy and safety of CRE combined with the bioavailability enhancer piperine (CREP), as a natural supplement for managing weight in obese individuals.

**Methods:**

A randomized, double-blind, placebo-controlled trial was conducted over 90 days involving 96 obese participants. Subjects received oral supplementation of 500 mg CRE with 5 mg piperine (CREP) twice daily, alongside a prescribed exercise and diet regimen. Primary outcomes included anthropometric measures such as body weight, body mass index, waist circumference, hip circumference, and waist–hip ratio. Secondary outcomes assessed included serum lipid parameters, Apolipoprotein B-100, calorie intake, physical activity, and safety evaluations.

**Results:**

CREP supplementation led to significant reductions in body weight, body mass index, waist, and hip circumference compared to placebo. Improvements in serum lipid profiles and Apolipoprotein B-100 levels were also observed in the CREP group. No adverse events were reported, and all laboratory parameters remained within normal ranges.

**Conclusion:**

The findings demonstrate that CREP is a safe and effective natural supplement for reducing abdominal obesity and improving lipid profiles. The observed benefits surpassed those of lifestyle management alone, supporting the potential development of CREP as a natural therapeutic option for weight management in obese individuals.

## 1. Introduction

Obesity has emerged as a major global public health concern and is ranked as the 5th leading cause of mortality worldwide, according to the World Health Organization.^[1]^ It is a key risk factor for a variety of chronic conditions, including type 2 diabetes mellitus, cardiovascular diseases, certain cancers, asthma, hypercholesterolemia, metabolic syndrome, hypertension, nonalcoholic fatty liver disease, osteoarthritis, depression, and chronic kidney disease.^[2–4]^ The Global Burden of Disease study in 2017 attributed 2.3 and 2.4 million deaths in men and women, respectively, to a body mass index (BMI) of ≥ 25 kg/m².^[5]^

The pathophysiology of obesity is multifactorial, involving complex interactions between genetic, dietary, environmental, lifestyle, and behavioral factors.^[6–8]^ Sedentary behavior and the consumption of calorie-dense diets are widely recognized as primary contributors to the global obesity epidemic.^[9]^ In recent years, the treatment landscape for obesity has rapidly evolved, with a focus on hormone-based therapies that target glucagon-like peptide 1 and glucose-dependent insulinotropic polypeptide pathways, which regulate fat mass and energy balance.^[10]^ However, many approved pharmaceutical options are associated with side effects such as nausea, constipation, headache, vomiting, insomnia, diarrhea, dizziness, elevation in heart rate, dry mouth, and sleep disorders.^[11]^

Due to these challenges, many plant-derived products have gained popularity for their potential to provide safe and effective alternatives for obesity management. Such natural compounds offer promising efficacy, low cost, and minimal adverse effects.^[12]^ Extensive research has demonstrated the ability of plant-derived compounds to modulate obesity-related mechanisms, including enhancing thermogenesis, suppressing appetite, reducing oxidative stress and systemic inflammation, inhibiting lipogenesis, promoting lipolysis, and decreasing lipid absorption.^[13–15]^

One plant of particular interest is *Cyperus rotundus* L. (commonly known as nut grass), a perennial herb from the Cyperaceae family. Traditionally, *C rotundus* has been used to treat a range of ailments, including gastrointestinal disorders, fever, obesity, inflammation, and skin diseases. Its bioactive constituents include stilbenes, flavonoids, quinones, saponins, alkaloids, and phenolic acids such as salicylic, protocatechuic, caffeic, and p-coumaric acids.^[16]^ Stilbenes, a group of naturally occurring phenolic compounds, are well-known for their health benefits, with resveratrol being the most studied example. Recent research has highlighted the potential of stilbenes like pterostilbene and piceatannol in obesity management.^[17,18]^ Scirpusin A and B, which are dimers of resveratrol and piceatannol, have shown promising pharmacological potential, though they remain less explored.^[18]^

*C rotundus* extract (CRE) was earlier shown to reduce adipogenesis in an experimental cell-based model in 3T3-L1 fibroblast cells. In a dose-dependent study, treatment of CRE reduced lipid accumulation with an IC_50_ value of 9.39 μg/mL without affecting cell viability. The adipogenesis reduction was also associated with reduced levels of transcripts for peroxisome proliferator-activated receptor γ and adipocyte protein 2/adipocyte fatty acid-binding protein. Further, in vivo study the antiobesity effect was analyzed in a high-fat diet-induced obese animal model (C57/BL6 mice). The animals fed with CRE resulted in a dose-dependent reduction in weight gain. A significant reduction of weight gain by 30% to 46% was observed with extract in high-fat diet-fed mice. The treatment showed a significant decrease in lipid parameters. The reduction in serum leptin and corticosterone levels was also observed indicating the effectiveness of CRE in weight management.^[19]^ In a previous pilot clinical study, we demonstrated that 525 mg of CRE, standardized to 5% stilbenes, and administered twice daily over 90 days, was safe and effective for managing obesity.^[19]^

In the current study, we investigate a formulation of CRE, standardized to contain 6% to 8% total stilbenes (scirpusin A, scirpusin B, and piceatannol) and combined with piperine, a bioavailability enhancer. Piperine enhances absorption by modulating p-glycoprotein-mediated drug and nutrient efflux, altering metabolic enzymes, and increasing intestinal absorption and thermogenesis.^[20]^ This study aims to evaluate the efficacy of the formulation (*Cyperus rotundus* extract with piperine [CREP]) in managing weight in obese adults.

## 2. Materials and methods

### 2.1. *C rotundus* extract

**Figure 1.**
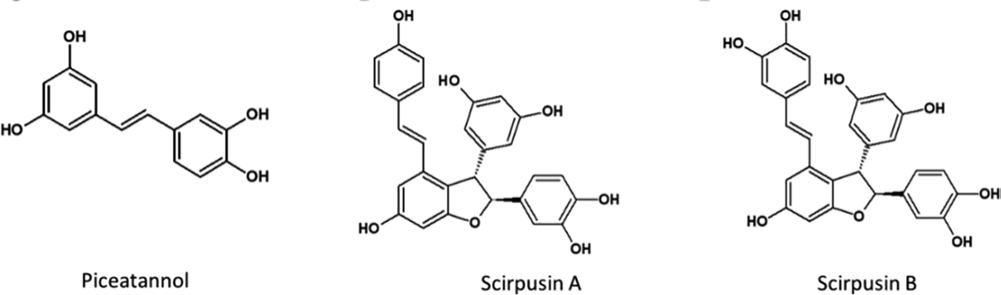
Structures of Piceatannol, Scirpusin A and Scirpusin B.

**Figure 2.**
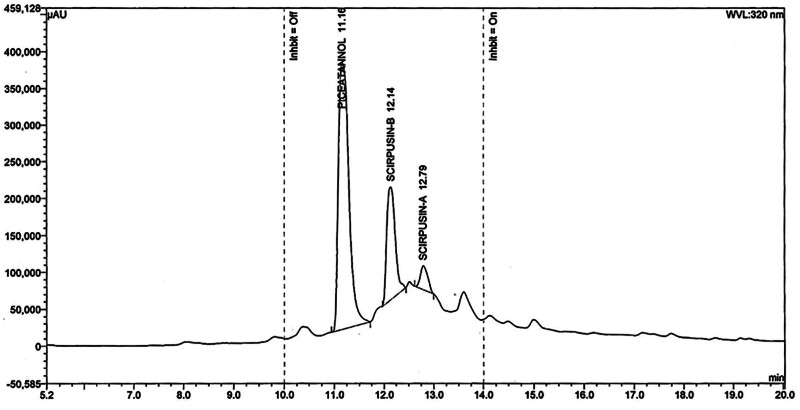
HPLC chromatogram of Scirpusin A, Scirpusin B, and Piceatannol. HPLC = high performance liquid chromatography.

CRE was obtained from the dried rhizomes, through a solvent extraction process. It was standardized to contain a minimum of 6% to 8% of total identified stilbenes scirpusin A, scirpusin B, and piceatannol (Fig. 1) by high performance liquid chromatography. BioPerine^®^ was prepared from the dried fruits of *Piper nigrum*, standardized to contain a minimum of 95% piperine. The high performance liquid chromatography chromatogram of scirpusin A, scirpusin B and piceatannol (detected at 320 nm), shown in Figure 2.

### 2.2. Study design and ethics

A randomized, double-blind, placebo-controlled study was conducted on obese participants from November 2022 to February 2023 at 2 centers in southern India. The study protocol (CW/109/CRP_OBES//II/JUL/22) was reviewed and approved by the Institutional Ethics Committee of Government Medical College and Government General Hospital, Srikakulam, India (EC Registration No: ECR/492/Inst/AP/2013/RR-20) and Bio Medical Research, Apollo First Med Hospitals,Chennai, India (EC Registration No: EC/NEW/INST/2020/527) before the initiation of the study. Written informed consent was taken from all the participants before enrollment. The study was conducted as per the Declaration of Helsinki, following Good Clinical Practice as required by the International Conference on Harmonization and all applicable local regulatory requirements. The trial was registered prospectively with the Clinical Trial Registry of India with the registration number CTRI/2022/09/045790 registered on 22/09/2022.

### 2.3. Study population

#### 2.3.1. Inclusion criteria

Male and female participants between the ages of 21 and 55 years with a BMI ≥ 30 kg/m^2^ and ≤ 40 kg/m². Participants were required to self-report a lack of physical activity and be willing to begin walking for 30 minutes daily for 5 days a week. Other inclusion criteria were the ability to swallow and retain oral medications, willingness to follow the recommended diet throughout the study, and agreement to attend follow-up visits.

#### 2.3.2. Exclusion criteria

Participants taking any drugs, having undergone weight loss surgery, adhering to a particular weight loss diet, following a special diet, engaging in exercise, or being diagnosed with pathophysiologic/genetic syndromes associated with obesity were excluded from the study. Other criteria for exclusion were a history of chronic smoking, excessive alcohol intake, evidence of malignancy, diabetes, hypertension, thyroid disease, lipid-lowering drugs, underlying inflammatory arthropathy, septic arthritis, inflammatory joint disease, gout, pseudo gout, Paget disease, joint fracture, acromegaly, fibromyalgia, Wilsons disease, Ochronosis, hemochromatosis, heritable arthritic disorder or collagen gene mutations or rheumatoid arthritis, coagulopathies, cardiovascular diseases, congestive heart failure, pancreatitis, lactic acidosis, hepatomegaly with steatosis, motor weakness, peripheral sensory neuropathy, psychiatric disorder, and severe pulmonary dysfunction. Participants diagnosed with an eating disorder, severe psychiatric disorders, weight loss in the last 6 months, prolonged medication with corticosteroids, antidepressants, anticholinergics, serious hepatic disorder, hypersensitivity to any of the herbal extracts or dietary supplements, pregnant or lactating women and females with a history of PCOS and participation in another clinical trial within the last 3 months were also excluded from the study.

### 2.4. Sample size

The sample size was calculated for a power of 80% and alpha = 0.05 significance level assuming a correlation of 0.14, based on the earlier published study. The required total sample size is 84 for evaluation. Allowing for 15% drop-out rate, the required sample size for recruitment is a total of 96 in 1:1 ratio between 2 treatment groups (i.e., 48 per treatment group).

#### 2.4.1. Randomization

Participants were randomized, using a predetermined block randomization schedule generated using computer-based randomization software (SAS 9.4, SAS Institute Inc. Panorama Consulting Group+2SAS+2, Cary). The randomization sequence was prepared by a statistician, independent of the sponsoring organization and not involved in the conduct or reporting of the study. An alpha code was generated for both the active and placebo to maintain blinding and concealment of allocations. A block randomization method with a block size of 6 was followed wherein the participants were randomized to receive either a placebo or an investigational product. The randomization codes were kept strictly confidential and were accessible only to authorized persons on an emergency basis as per the standard operating procedures until the time of unblinding.

#### 2.4.2. Intervention

The study participants received 500 mg CRE with 5 mg piperine (CREP) or a placebo containing microcrystalline cellulose capsules twice daily (see Table S1, Supplemental Digital Content, https://links.lww.com/MD/Q564). Capsules were administered after food for 90 days. The number of capsules dispensed to the participants and returned at each visit was recorded in the case report file. All the enrolled participants were asked to initiate lifestyle changes (a healthy diet with cardio/exercise for 5 days a week for at least 30 minutes) along with placebo or CREP supplements.

#### 2.4.3. Efficacy measures – primary and secondary

Participants underwent a medical and physical examination at every visit. The primary outcome measures were changes in body weight, BMI, waist and hip circumference, and waist-to-hip ratio (WHR) from day 0 to day 90. The secondary outcome measures were change in serum lipid profiles of low-density lipoprotein cholesterol (LDL-C), very low-density lipoprotein cholesterol (VLDL-C), high-density lipoprotein cholesterol (HDL-C), total cholesterol (TC), and triglycerides (TG) from day 0 to day 90. The change in Apolipoprotein B-100 (Apo B) levels in serum, total number of calories intake, and minutes of physical activity were also assessed as a secondary outcome. Safety assessments included monitoring any adverse event throughout the study and laboratory hematological, biochemical parameters (total bilirubin, alkaline phosphatase, alanine aminotransferase/serum glutamic pyruvic transaminase, aspartate aminotransferase/serum glutamic oxaloacetic transaminase, thyroid, and renal), and urinary parameters.

### 2.5. Measurements

Body weight was taken in a surgical gown without footwear and other external apparel, using a weighing balance of the same make and model at each site. The waist circumference (WC) was measured by placing the measuring tape horizontally around the waist just above the hip bone and height was measured using a stadiometer.

### 2.6. Biochemical and biomarker evaluation

The serum biochemical parameters were estimated in Government Medical College and Government General Hospital (Srikakulam), Apollo First Med Hospitals (Chennai) using ADVIA 1800 (Siemens Healthineers India, Mumbai, India) and DxH 900 (Beckman Coulter, Brea). Biomarker Apo B were estimated by enzyme linked immunosorbent assay, using commercial kits (Roche Diagnostics GmbH, Penzberg, Bavaria, Germany; bayern-international.de+2roche.com+2) at Suburban Diagnostics, India as per the manufacturer’s instructions.

### 2.7. Exercise and calorie intake measurement

The participants in both the placebo and CREP group were instructed to undertake at least 30 minutes of physical exercise (brisk walking) and avoid processed and fried food, during the study period along with CREP or placebo supplements. The calorie intake and exercise time were monitored using a patient diary. The participants recorded the food consumed every day and the exercise time every day in the diary. Nutrition charts were used to convert the food into calories consumed per day.^[21]^

### 2.8. Statistical analysis

For continuous variables and normally distributed data within the group were compared using paired *t*-test, and data were presented as mean difference and *P*-value. Normality was checked by Shapiro–Wilk test. The non-normally distributed data within the group were compared using the Wilcoxon Signed Rank test, and the data were presented as median, inter-quartile range, and *P*-value. Repeated measures analysis of variance was performed for measured variables to evaluate the within-group change from baseline.

An unpaired *t*-test was used for the comparative analysis between treatment groups for normally distributed data and the Mann–Whitney *U* test was used for non-normally distributed data and a *P*-value was presented. The level of statistical significance was defined as *P* < .05. The frequency and percentage of the population are presented as categorical variables. A descriptive comparison is provided to differentiate the treatment effect between the treatment groups and within treatment groups.

## 3. Results

### 3.1. Demographic and baseline characteristics

**Figure 3.**
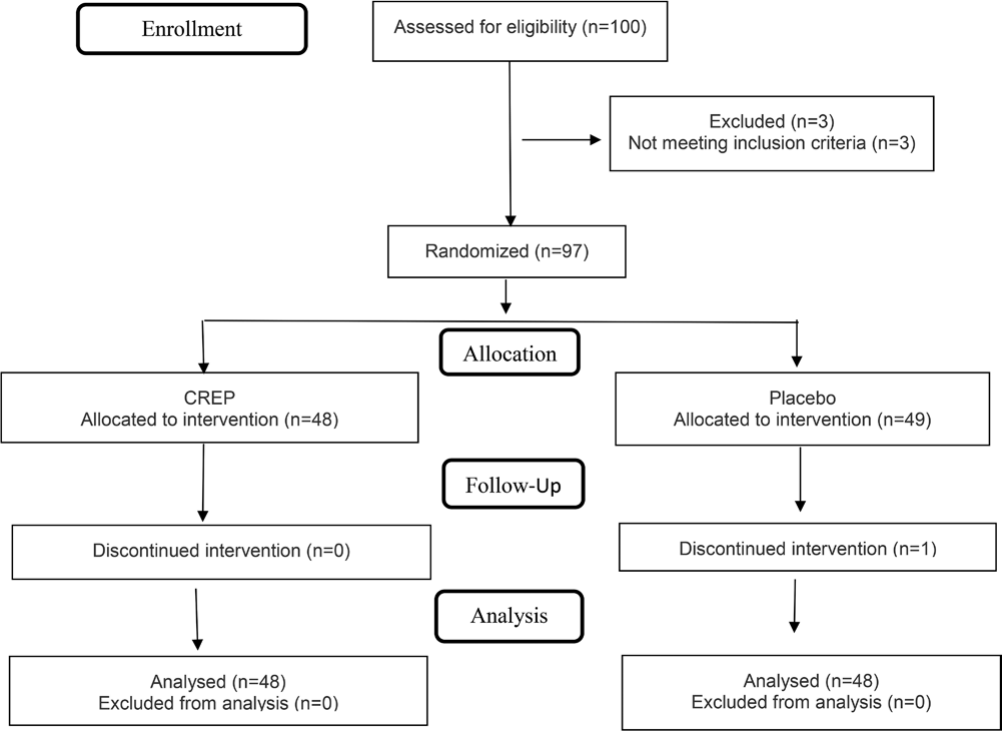
Consort flow diagram. CREP = *Cyperus rotundus* extract with piperine.

In total, 100 participants were screened, and 97 were randomized to receive either CREP (N = 48, 29 male and 19 female) or placebo groups (N = 49, 23 male and 26 female). In the placebo group 1 participant discontinued the study (Fig. 3).

All the participants were Indian and had no history of smoking, drug abuse, or alcoholism. In the CREP the average age was 39.25 years; height was 158.98 cm; weight was 82.75 kg; and BMI was 32.98 kg/m^2^ whereas in placebo the overall average age was 38.25 years; height was 159.33 cm; weight was 82.79 kg; and BMI was 32.56 kg/m^2^. The demographic and baseline characteristics of the participants were comparable in the 2 groups as shown in Table 1.

**Table 1:** Demographic and baseline characteristics.

Characteristics	CREP (N = 48)	Placebo (N = 48)	*P* value
Age (yr)	39.25 ± 8.59	38.25 ± 9.10	.58
Gender			
Male, N (%)	29 (60.42)	23 (47.92)	.22
Female, N (%)	19 (39.58)	25 (52.08)	
Height (cm)	158.98 ± 7.46	159.33 ± 8.14	.82
Weight (kg)	82.75 ± 8.74	82.79 ± 8.38	.98
Body mass index (kg/m^2^)	32.98 ± 2.30	32.56 ± 1.60	.32
Body temperature (^o^C)	97.97 ± 0.72	98.01 ± 0.63	.44
Systolic blood pressure (mm Hg)	119.27 ± 7.09	120.75 ± 6.63	.18
Diastolic blood pressure (mm Hg)	81.15 ± 5.85	81.56 ± 5.73	.15
Pulse rate (bpm)	78.44 ± 4.98	78.40 ± 5.12	.22

Values are expressed as Mean ± SD, values are given for the baseline demographics.

CREP = *Cyperus rotundus* extract with Piperine.

### 3.2. Primary endpoints

#### 3.2.1. Anthropometric parameters

**Figure 4.**
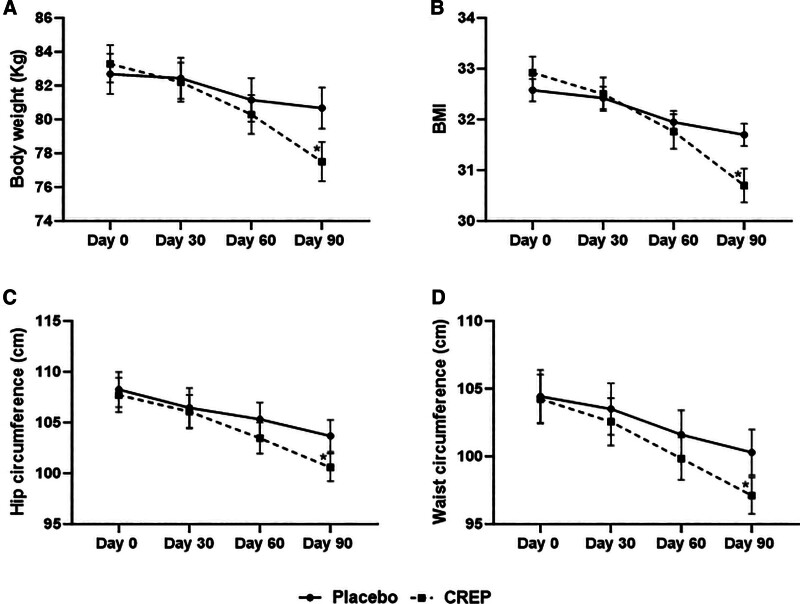
Changes in anthropometric parameters: (A) body weight, (B) body mass index, (C) hip circumference, and (D) waist circumference in placebo and CREP group. Values are expressed as Mean ± SEM. Repeated measure ANOVA (*P* value) and Dunnett Multiple Comparison Test. (*) were performed to evaluate the within-group change from baseline. ANOVA = analysis of variance, BMI = body mass index, CREP = *Cyperus rotundus* extract with piperine.

The change in body weight, BMI, hip circumference, WC, and the WHR was measured during each visit and are presented in Figure 4. Both body weight (83.28 ± 7.73 to 77.50 ± 8.01 vs 82.69 ± 8.30 to 80.67 ± 8.45, *P* < .0001) and BMI (32.92 ± 2.27 to 30.70 ± 2.32 vs 32.58 ± 1.57 to 31.70 ± 1.52, *P* < .0001) decreased in CREP group at the end of the study compared to placebo (Table 2). After 30 days of treatment, a reduction in body weight (1.4% vs 0.5%) was observed whereas after 60 days it showed a reduction of 3.7% and 2.1% from baseline in the treatment and placebo groups, respectively. However, at the end of 90 days, the reduction of body weight from baseline for the treatment and placebo groups was 7.1% and 2.6% respectively. The mean BMI of both the groups was reduced during the study (Table 2). After 60 and 90 days of treatment, the mean reduction from baseline of 3.74% and 6.9% respectively was observed in the treatment group, which was statistically significant compared with the placebo group (60 days, 1.49%; 90 days, 2.7%). Thus, the body weight and BMI showed a significant difference from day 0 to day 90 in participants supplemented with CREP but not in the placebo group. Further, the waist and hip circumference showed a reduction in the CREP group after 90 days of treatment. The CREP showed a reduction in both WC from 104.23 ± 12.57 to 97.11 ± 9.38 cm and hip circumference from 107.71 ± 11.85 to 100.58 ± 9.59 cm compared to placebo. Though not significant, the WHR of participants treated with CREP was found to be better in therapeutic response from day 0 to day 90 compared to placebo.

**Table 2:** Mean change in anthropometric measurements.

Parameter	Group	Day 0	Day 90	Mean change	*P* value
Body weight (kg)	CREP (N = 48)	83.28 ± 7.73	77.50 ± 8.01***	−5.85	<.0001
	Placebo (N = 48)	82.69 ± 8.30	80.67 ± 8.45	−2.02	
BMI (kg/m^2^)	CREP (N = 48)	32.92 ± 2.27	30.70 ± 2.32***	−2.23	<.0001
	Placebo (N = 48)	32.58 ± 1.57	31.70 ± 1.52	−0.88	
Waist circumference (cm)	CREP (N = 48)	104.23 ± 12.57	97.11 ± 9.38*	−7.11	.0013
	Placebo (N = 48)	104.43 ± 13.52	100.29 ± 11.69	−4.13	
Hip circumference (cm)	CREP (N = 48)	107.71 ± 11.85	100.58 ± 9.59**	−7.13	.0004
	Placebo (N = 48)	108.25 ± 12.01	103.68 ± 10.97	−4.57	
Waist–hip ratio	CREP (N = 48)	0.96 ± 0.04	0.96 ± 0.03	0.00	.8312
	Placebo (N = 48)	0.96 ± 0.05	0.96 ± 0.04	0.00	

BMI = body mass index, CREP = *Cyperus rotundus* extract with piperine.

Values are expressed as Mean ± SD.

* *P* < .05. *P* value indicates the significance of change between the placebo and CREP group.

** *P* < .001.

*** *P* < .0001.

### 3.3. Secondary endpoints

#### 3.3.1. Serum lipid profile and Apolipoprotein B biomarker

The changes in serum lipid parameters, such as TC, TG, LDL-C, HDL-C, and VLDL-C at baseline (day 0) and end of the study (day 90) between the groups are presented in Table 3. The TC (196.00 ± 35.12 to 177.5 ± 34.02 vs 191.88 ± 39.71 to 183.19 ± 40.47) and TG (174.60 ± 62.18 to 139.08 ± 46.92 vs 165.52 ± 64.59 to 150.27 ± 51.37) showed a significant reduction from day 0 to day 90 in CREP group (0.002 and 0.006 respectively) when compared to placebo.

**Table 3:** Mean changes in serum lipid parameters and Apolipoprotein B.

Parameter	Group	Day 0	Day 90	Mean change	*P* value
TC (mg/dL)	CREP (N = 48)	196.00 ± 35.12	177.5 ± 34.02*	−18.50	.0022
	Placebo (N = 48)	191.88 ± 39.71	183.19 ± 40.47	−8.69	
HDL-C (mg/dL)	CREP (N = 48)	41.58 ± 7.77	45.27 ± 8.00	3.69	.1192
	Placebo (N = 48)	41.48 ± 7.27	43.69 ± 6.09	2.21	
LDL-C (mg/dL)	CREP (N = 48)	128.33 ± 31.34	121.25 ± 29.10	−7.08	.4210
	Placebo (N = 48)	124.73 ± 31.43	120.77 ± 27.50	−5.53	
VLDL-C (mg/dL)	CREP (N = 48)	23.40 ± 8.06	20.88 ± 8.57	−2.52	.4461
	Placebo (N = 48)	22.23 ± 8.59	20.08 ± 7.24	−2.15	
TG (mg/dL)	CREP (N = 48)	174.60 ± 62.18	139.08 ± 46.92*	−35.52	.0062
	Placebo (N = 48)	165.52 ± 64.59	150.27 ± 51.37	−15.25	
Apo B	CREP (N = 48)	73.42 ± 19.80	55.04 ± 18.34	−18.39	.5438
	Placebo (N = 48)	74.7 ± 21.49	52.81 ± 18.49	−21.89	

Values are expressed as Mean ± SD.

Apo B = Apolipoprotein B, BMI = body mass index, CREP = *Cyperus rotundus* extract with piperine, HDL-C = high-density lipoprotein cholesterol, LDL-C = low-density lipoprotein cholesterol, TC = total cholesterol, TG = triglycerides, VLDL-C = very low-density lipoprotein cholesterol.

* *P* value < .05 indicates the significance of change between the placebo and CREP group. Significance difference from day 0 to day 90 within the placebo and CREP group.

LDL-C (128.33 ± 31.34 to 121.25 ± 29.10 vs 124.73 ± 31.43 to 120.77 ± 27.50) and VLDL-C (23.40 ± 8.06 to 20.88 ± 8.57 vs 22.23 ± 8.59 to 20.08 ± 7.24) showed a reduction in CREP group from day 0 to day 90 but it was not significant when compared to placebo. Further, an increase in HDL-C (41.58 ± 7.77 to 45.27 ± 8.00 vs 41.48 ± 7.27 to 43.69 ± 6.09) was observed in both CREP and placebo groups from day 0 to day 90 but the change between CREP and placebo was not significant.

Studies have reported that Apo B acts as a biomarker for cardiovascular diseases, and metabolic syndrome. The CREP group showed a significant decrease in the mean of biomarker Apo B (73.42 ± 19.80 to 55.04 ± 18.34) from day 0 to day 90 (Table 3).

### 3.4. Calorie intake and physical activity

All the participants maintained a calorie intake of 1200 to 1400 calories per day and exercised for an average of 40 to 50 minutes per day throughout the study (Table 4).

**Table 4:** Mean change in average calorie intake and duration of physical activity per day.

Parameter	Group	Month 1	Month 2	Month 3	*P* value
Calorie intake	CREP (N = 48)	1270 ± 466.3	1256 ± 456.3	1243 ± 442.5	.85
	Placebo (N = 48)	1320 ± 466.2	1294 ± 473.7	1270 ± 469.1	
Minutes of physical activity	CREP (N = 48)	45.58 ± 10.88	44.46 ± 9.82	44.54 ± 11.47	.37
	Placebo (N = 48)	48.07 ± 14.96	45.06 ± 12.93	44.79 ± 11.11	

The average calorie intake per day and average minutes of physical exercise undertaken by the participants are represented as Mean ± Standard deviation. *P* values were computed by two-way ANOVA.

ANOVA = analysis of variance, CREP = *Cyperus rotundus* extract with piperine.

### 3.5. Safety parameters

Safety analysis was performed for all the participants who completed the study. There was no significant change in safety parameters within the treatment groups in any of the study visits. All the laboratory parameters like biochemical, hematological, and urine analysis were found to be within the specified range throughout the study (see Tables S2–S4, Supplemental Digital Content, https://links.lww.com/MD/Q564). Mild adverse events were reported by a few participants such as headache, fever, leg pain, back pain, and stomach upset (Table 5).

**Table 5:** List of adverse events and their resolution time.

Gender	Group	Adverse effect	Resolved time	Outcome	Severity
Male	CREP	Headache	30 min	Resolved	Mild
Female	Placebo	Leg pain	30 min	Resolved	Mild
Female	CREP	Leg pain	3 h 30 min	Resolved	Mild
Female	CREP	Headache	30 min	Resolved	Mild
Male	Placebo	Headache	1 h 30 min	Resolved	Mild
Male	Placebo	Fever	9 h	Resolved	Mild
Female	Placebo	Stomach upset	30 min	Resolved	Mild
Female	Placebo	Headache	1 h 10 min	Resolved	Mild
Male	CREP	Back pain	2 h	Resolved	Mild
Male	CREP	Headache	2 h	Resolved	Mild
Male	CREP	Headache	2 h	Resolved	Mild
Male	Placebo	Headache	1 h 30 min	Resolved	Mild
Male	CREP	Headache	2 h 30 min	Resolved	Mild
Male	Placebo	Back pain	5 h	Resolved	Mild
Male	CREP	Fever	3 h	Resolved	Mild

All the adverse effects were resolved, and intervention continued without any changes.

CREP = *Cyperus rotundus* extract with piperine.

Mild adverse events were resolved during the study period and were not related to the study medication. No participants discontinued the study due to adverse effects. These results suggest that CREP is safe and effective for weight loss in obese participants.

## 4. Discussion

In the present study, we observed that CRE containing 6% to 8% stilbenes (scirpusin A, scirpusin B, and piceatannol) with piperine (CREP) was effective in reducing body weight, BMI, WC, hip circumference, and improved the serum lipid and Apo B levels. The lifestyle changes induced a reduction in body weight and lipid levels in the placebo and CREP supplementation resulted in an improvement beyond lifestyle management.

Obesity, a complex metabolic disorder, is defined by excessive fat accumulation resulting from energy intake exceeding energy expenditure, which in turn impairs overall health. Fat distribution in the body typically occurs in subcutaneous adipose tissue and visceral adipose tissue, each with distinct metabolic properties. Visceral fat, which accumulates predominantly around the abdominal area, also known as central or abdominal obesity, has been shown to pose greater health risks than overall body fat. Abdominal obesity is strongly associated with increased susceptibility to chronic diseases, including cardiovascular diseases and metabolic syndrome.^[22–25]^

Various techniques such as BMI, WC, WHR, and body composition assessment tools like dual-energy X-ray absorptiometry and computed tomography are employed to evaluate visceral fat distribution.^[26,27]^ While BMI remains widely used in both clinical and research settings, it is limited by its inability to specify fat location.^[28,29]^ On the other hand, measurements like WC and WHR are commonly used in clinical practice as they provide estimates of fat distribution.^[30]^ Specifically, WC reflects both visceral and subcutaneous fat, while hip circumference predominantly represents subcutaneous fat.^[29]^ An elevated WHR indicates a higher proportion of intra-abdominal fat, which correlates with increased risk for obesity-related diseases.^[31,32]^ Given their reliability, WC and WHR are frequently used as surrogates for visceral adiposity and to assess the risk of cardiovascular diseases and metabolic syndrome.^[32–34]^

In the current study, participants receiving CREP supplementation for 90 days demonstrated reductions in body weight (7.1%), BMI (6.9%), WC (6.8%), and hip circumference (6.6%), suggesting a positive effect on abdominal obesity. Weight loss of 5% or more is considered clinically meaningful for improving metabolic health,^[35]^ and in this study, 43 of 48 participants (89.5%) in the CREP group achieved this threshold, compared to only 6 of 48 participants (12.5%) in the placebo group. This highlights the significant impact of CREP on body weight reduction.

Dyslipidemia, characterized by abnormal lipid metabolism resulting in elevated TG, LDL-C, VLDL-C, and reduced HDL-C, is common in obesity. The excessive release of free fatty acids from visceral fat can lead to increased hepatic synthesis of LDL and VLDL, raising plasma lipid levels.^[36]^ In this study, CREP supplementation led to more significant improvements in lipid parameters compared to placebo, with reductions in TC, LDL-C, VLDL-C, and TG levels, alongside an increase in HDL-C levels, suggesting beneficial effects on dyslipidemia. Although not all lipid reductions reached statistical significance, lifestyle changes implemented by both groups may have contributed to these results.

Apo B is a key marker for circulating atherogenic lipoproteins, offering a more precise indicator of cardiovascular risk than traditional cholesterol measures.^[37–40]^ Our findings revealed a significant reduction in Apo B levels in the CREP group, indicating improved lipid metabolism and a reduced risk of cardiovascular disease. This reduction in Apo B, in conjunction with improved lipid profiles, may be attributed to CREP’s impact on fat metabolism, which warrants further investigation.

Notably, both the CREP and placebo groups reported similar caloric intake and physical activity levels, suggesting that CREP’s effects were not mediated through appetite suppression or changes in energy intake. The placebo group did experience weight and lipid reductions, likely attributable to lifestyle interventions, underscoring the combined effects of lifestyle and supplementation in managing obesity.

Previous studies have demonstrated the anti-obesity potential of CRE, particularly its stilbenoid components (piceatannol, scirpusins A and B).^[19]^ In this study, the stilbenoid content was optimized to 6% to 8%, and piperine was added to enhance bioavailability. Stilbenoids have been shown to inhibit lipid absorption, suppress adipogenesis, and boost energy metabolism, thus aiding in weight management.^[41–46]^ Piperine, in particular, is recognized for enhancing the absorption of bioactive compounds, including those in CRE.^[20,47]^

Our findings align with previous studies on the anti-adipogenic effects of CRE.^[19]^ Notably, this study demonstrated greater reductions in visceral fat compared to earlier trials, despite less overall weight loss. The enhanced effect on visceral obesity may be linked to CREP’s modulation of lipid metabolism, as indicated by its impact on cholesterol and Apo B levels.^[48]^ Preclinical studies suggest that CRE may increase the expression of uncoupling protein 1, a key regulator of thermogenesis and fat metabolism^[49]^ though this requires further validation in human studies.

The safety and tolerability of CREP were confirmed over the 90-day study period, with no serious adverse events reported. Mild adverse events were observed in both the CREP and placebo groups but were resolved during the study and were unrelated to the treatment.

The study’s limitations include the lack of direct body composition measurements and the absence of adipokine and inflammatory marker assessments. Additionally, participants were not followed up post-study to assess the sustainability of weight loss. Future research should involve long-term studies, including biomarker analyses and follow-up data, to further elucidate the mechanisms of CREP and its long-term efficacy in weight management.

## 5. Conclusion

CREP was found to be safe, well-tolerated, and effective in managing obesity by reducing central obesity and lowering circulating lipid levels. The combination of lifestyle modifications, including a healthy diet and a minimum of 30 minutes of physical activity per day, along with CREP supplementation, led to greater reductions in body weight and cholesterol levels compared to lifestyle changes alone. Future long-term studies across diverse ethnic populations will be valuable in establishing the role of CREP as a supplement for weight management and in reducing obesity-related cardiovascular risk in obese individuals.

## Acknowledgments

We would like to thank the entire clinical research team from Apollo First Med Hospitals (Chennai) and Government Medical College and Government General Hospital (Srikakulam) for their assistance. The authors thank all the study participants and clinical coordinators from the 2 centers for their support. The study was supported by Sami-Sabinsa Group Limited and was conducted independently by principal investigators.

## Author contributions

**Conceptualization:** Anju Majeed, Shaheen Majeed.

**Data curation:** Lakshmi Mundkur.

**Investigation:** T. V. Devarajan, S. S. V. V. Narasinga Rao.

**Methodology:** Satish Gudimallam, Manjunatha Ramanujappa, Lakshmi Mundkur.

**Project administration:** Satish Gudimallam, Manjunatha Ramanujappa.

**Resources:** Anju Majeed, Shaheen Majeed.

**Supervision:** T. V. Devarajan, S. S. V. V. Narasinga Rao.

**Validation:** Lakshmi Mundkur.

**Writing – original draft:** Smitha Thazhathidath.

**Writing – review & editing:** Anju Majeed, Shaheen Majeed, T. V. Devarajan, S. S. V. V. Narasinga Rao, Satish Gudimallam, Manjunatha Ramanujappa, Smitha Thazhathidath, Lakshmi Mundkur.

## Supplementary Material


