# Supplementary material for: Efficacy and safety of Cyperus rotundus extract on weight management in obese individuals: A randomized, double-blind, placebo-controlled study
Source: Medicine (Baltimore). 2025 Nov 21;104(47):e45666. doi: 10.1097/MD.0000000000045666 (PMC12643649; doi:10.1097/MD.0000000000045666)
Supplement: Supplementary file 1 [file medi-104-e45666-s001.docx]

**Table S1: Composition of Investigational Product (CREP) and Placebo**

**Label Claim**

**Active: Stilbenoid Extract + Piperine (CREP)**

Stilbenoids (6 to 8%) 41.35 mg /capsule

Piperine (95%) 5.97 mg / capsule

Average weight of capsule = 594.37 mg

**Placebo: Microcrystalline Cellulose**

CREP: *Cyperus rotundus* extract with Piperine

**Table S2: Biochemical parameters**

| **Parameter** | **CREP** | | **Placebo** | | **CREP *vs.* Placebo**  **P value** |
| --- | --- | --- | --- | --- | --- |
|  | **Day 0** | **Day 90** | **Day 0** | **Day 90** |  |
| Total Bilirubin (mg/dL) | 0.70 ± 0.19 | 0.71 ± 0.17 | 0.65 ± 0.16 | 0.69 ± 0.17 | 0.117 |
| Alkaline phosphatase (IU/L) | 74.90 ± 16.55 | 74.31 ± 17.60 | 69.63 ± 15.00 | 69.92 ± 15.26 | 0.447 |
| AST/SGOT (U/L) | 27.52 ± 9.73 | 27.79 ± 8.32 | 25.92 ± 10.59 | 26.79 ± 9.09 | 0.513 |
| ALT/SGPT (U/L) | 31.29 ± 11.44 | 30.71 ± 9.65 | 29.10 ± 13.71 | 29.44 ± 11.11 | 0.134 |
| FBS (mg/dL) | 95.15 ± 10.07 | 92.56 ± 6.63 | 93.5 ± 9.21 | 91.65 ± 7.1 | 0.792 |
| HbA1C (%) | 5.49 ± 0.37 | 5.35 ± 0.37 | 5.39 ± 0.42 | 5.37 ± 0.41 | 0.011 |
| Serum creatinine (mg/dL) | 0.84 ± 0.16 | 0.83 ± 0.15 | 0.79 ± 0.16 | 0.80 ± 0.14 | 0.114 |
| BUN | 8.12 ± 2.26 | 8.43 ± 2.78 | 8.38 ± 3.56 | 8.71 ± 3.53 | 0.792 |
| TSH (mIU/L) | 1.99 ± 1.04 |  | 2.07 ± 0.99 |  |  |
| T3 (ng/mL) | 96.77 ± 23.29 |  | 96.48 ± 20.00 |  |  |
| T4 (µg/mL) | 5.04 ± 3.78 |  | 5.07 ± 3.74 |  |  |

CREP: *Cyperus rotundus* extract with Piperine; AST/SGOT: Aspartate Aminotransferase/Serum Glutamic Oxaloacetic Transaminase; ALT/SGPT: Alanine Aminotransferase/Serum Glutamic Pyruvic Transaminase; FBS: Fasting Blood Sugar; HbA1C: Glycosylated Hemoglobin; BUN: Blood Urea Nitrogen; TSH: Thyroid Stimulating Hormone.

Values are expressed as Mean ± SD. No clinically significant difference was observed in the biochemical parameters between Day 0 and Day 90 in both CREP and placebo groups. The P value of the inter group statistical analysis was also

not significant.

**Table S3: Hematological parameters**

| **Parameter** | **CREP** | | **Placebo** | | **CREP *vs.*Placebo**  **P value** |
| --- | --- | --- | --- | --- | --- |
|  | **Day 0** | **Day 90** | **Day 0** | **Day 90** |  |
| Hb (gm) | 13.24 ± 1.62 | 13.71 ± 1.51 | 12.93 ± 1.74 | 13.63 ± 1.87 | 0.578 |
| RBC (million/mcl) | 4.44 ± 0.84 | 4.46 ± 0.90 | 4.23 ± 0.66 | 4.41 ± 0.84 | 0.107 |
| Platelet count (lakhs/cu mm) | 2.82 ± 0.72 | 2.82 ± 0.70 | 2.68 ± 0.59 | 2.71 ± 0.56 | 0.892 |
| Packed cell volume (%) | 40.29 ± 4.66 | 41.36 ± 4.39 | 39.4 ± 4.76 | 40.92 ± 4.83 | 0.301 |
| Mean cell volume (FL) | 85.94 ± 5.53 | 85.58 ± 4.93 | 85.13 ± 6.08 | 85.40 ± 5.87 | 0.287 |
| Mean platelet volume (FL) | 8.07 ± 0.70 | 8.06 ± 0.71 | 8.08 ± 0.71 | 7.85 ± 0.75 | 0.012 |
| Mean corpuscular Hb (pg) | 27.98 ± 1.87 | 27.42 ± 1.75 | 27.94 ± 2.11 | 28.13 ± 1.78 | 0.534 |
| Mean corpuscular Hb concentration (g/dL) | 32.90 ± 1.40 | 32.71 ± 1.38 | 32.75 ± 1.52 | 32.83 ± 1.74 | 0.537 |
| Total leukocyte count (cell/cm^2^) | 8074.79 ± 1673.06 | 8331.04 ± 1608.49 | 7546.25 ± 1431.11 | 7791.25 ± 1385.92 | 0.895 |
| ESR | 9.46 ± 5.70 | 6.56 ± 6.11 | 9.92 ± 6.44 | 8.09 ± 5.38 | 0.141 |
| Lymphocytes (%) | 30.60 ± 4.24 | 30.31 ± 3.20 | 30.21 ± 3.72 | 30.29 ± 6.58 | 0.679 |
| Eosinophils (%) | 4.31 ± 1.89 | 4.25 ± 2.02 | 5.02 ± 2.36 | 4.77 ± 1.75 | 0.773 |
| Monocytes (%) | 4.42 ± 1.46 | 4.69 ± 1.36 | 4.85 ± 2.07 | 4.94 ± 1.59 | 0.553 |
| Neutrophils (%) | 60.60 ± 4.54 | 60.65 ± 3.28 | 59.81 ± 3.81 | 59.90 ± 6.47 | 0.181 |
| Basophils (%) | 0.06 ± 0.24 | 0.10 ± 0.31 | 0.10 ± 0.31 | 0.10 ± 0.31 | 0.419 |

CREP: *Cyperus rotundus* extract with Piperine; Hb: Hemoglobin; RBC: Red Blood Cells; ESR: Erythrocyte Sedimentation Rate.

Values are expressed as Mean ± SD. No clinically significant difference was observed in the hematological parameters between Day 0 and Day 90 in both CREP and placebo groups.

The P value of the inter group statistical analysis was also not significant.

**Table S4: Urine analysis**

| **Parameter** | **CREP** | | **Placebo** | | **CREP *vs.* Placebo**  **P value** |
| --- | --- | --- | --- | --- | --- |
|  | **Day 0** | **Day 90** | **Day 0** | **Day 90** |  |
| **Urine pH** | 6.11 ± 0.77 | 6.20 ± 0.74 | 6.06 ± 0.73 | 6.23 ± 0.65 | 0.866 |
| **Specific gravity** | 1.02 ± 0.01 | 1.02 ± 0.01 | 1.02 ± 0.01 | 1.02 ± 0.01 | 0.340 |

CREP: *Cyperus rotundus* extract with Piperine.

Values are expressed as Mean ± SD. No clinically significant difference was observed in the urinary parameters between Day 0 and Day 90 in both CREP and placebo groups.

The P value of the inter group statistical analysis was also not significant.
